# Estrogen-induced DNA synthesis in vascular endothelial cells is mediated by ROS signaling

**DOI:** 10.1186/1471-2261-6-16

**Published:** 2006-04-11

**Authors:** Quentin Felty

**Affiliations:** 1 Department of Environmental & Occupational Health, Robert Stempel School of Public Health, Florida International University, Miami, FL 33199, USA

## Abstract

**Background:**

Since estrogen is known to increase vascular endothelial cell growth, elevated estrogen exposure from hormone replacement therapy or oral contraceptives has the potential to contribute in the development of abnormal proliferative vascular lesions and subsequent thickening of the vasculature. How estrogen may support or promote vascular lesions is not clear. We have examined in this study whether estrogen exposure to vascular endothelial cells increase the formation of reactive oxygen species (ROS), and estrogen-induced ROS is involved in the growth of endothelial cells.

**Methods:**

The effect of estrogen on the production of intracellular oxidants and the role of estrogen-induced ROS on cell growth was studied in human umbilical vein endothelial cells. ROS were measured by monitoring the oxidation of 2'7'-dichlorofluorescin by spectrofluorometry. Endothelial cell growth was measured by a colorimetric immunoassay based on BrdU incorporation into DNA.

**Results:**

Physiological concentrations of estrogen (367 fmol and 3.67 pmol) triggered a rapid 2-fold increase in intracellular oxidants in endothelial cells. E2-induced ROS formation was inhibited to basal levels by cotreatment with the mitochondrial inhibitor rotenone (2 μM) and xanthine oxidase inhibitor allopurinol (50 μM). Inhibitors of NAD(P)H oxidase, apocynin and DPI, did not block E2-induced ROS formation. Furthermore, the NOS inhibitor, L-NAME, did not prevent the increase in E2-induced ROS. These findings indicate both mitochondria and xanthine oxidase are the source of ROS in estrogen treated vascular endothelial cells. E2 treated cells showed a 2-fold induction of BrdU incorporation at 18 h which was not observed in cells exposed to vehicle alone. Cotreatment with ebselen (20 μM) and NAC (1 mM) inhibited E2-induced BrdU incorporation without affecting the basal levels of DNA synthesis. The observed inhibitory effect of NAC and ebselen on E2-induced DNA synthesis was also shown to be dose dependent.

**Conclusion:**

We have shown that estrogen exposure stimulates the rapid production of intracellular ROS and they are involved in growth signaling of endothelial cells. It appears that the early estrogen signaling does not require estrogen receptor genomic signaling because we can inhibit estrogen-induced DNA synthesis by antioxidants. Findings of this study may further expand research defining the underlying mechanism of how estrogen may promote vascular lesions. It also provides important information for the design of new antioxidant-based drugs or new antioxidant gene therapy to protect the cardiovascular health of individuals sensitive to estrogen.

## Background

In contrast to previous findings indicating cardioprotective effects of estrogen [1], recent primary and secondary trials of combined oral contraceptives and hormone replacement therapy have found no benefit for coronary heart disease, instead they showed an increase risk for stroke and venous thrombosis [2]. Estrogen increases inflammation and it may trigger coronary events in advanced atherosclerotic lesions [3]. At the average age of menopause, a substantial proportion of women have elevated atherosclerotic lesions, and a smaller proportion already has advanced lesions. The use of synthetic estrogens can produce thromboembolic disorders. An increased incidence of deep vein phlebitis and pulmonary embolism has been reported in young women who use oral contraceptives [4]. Intracranial venous thrombosis and secondary increases in the risk of stroke have also been noted. In experimental animal studies, estrogen has been shown to promote stroke in hypertensive rats [5], produce severe degenerative atherosclerotic effects on coronary arteries [6], and increase susceptibility to early atherosclerosis in male mice via the estrogen receptor-(ER) α [7]. Human studies have implicated the dysregulation of the ERα signaling pathway in the development of cardiovascular disease in men. The Framingham Heart Study showed that a higher risk of myocardial infarction was common to males with ERα gene (ESR1) variant [8]. Men with the ESR1 variant also showed more complex atherosclerotic plaque pathology [9]. Whether there is an association of myocardial infarction in women with the ESR1 variant and if there is a significant interaction with elevated estrogen exposure has yet to be determined. These findings suggest that estrogen is harmful to the cardiovascular system, but how exposure to excess or elevated level of estrogen produces adverse effects to the cardiovascular system is not clear.

Elevated estrogen exposure is known to increase inflammation [10] which is implicated in the development of vascular lesions [11]. Advanced atherosclerotic lesions are characterized by abnormal cell proliferation that can lead to vascular blockage, myocardial infarction, and stroke [12]. Although several different cell types, including vascular smooth muscle cells, inflammatory cells, and fibroblasts are involved in this vasculoproliferative process; we recognize endothelial cells to be the initial site of injury because it reacts with physical and chemical stimuli within the circulation. Atherosclerotic lesions have been proposed to occur as a result of the monoclonal expansion of a mutated vascular cell [13]. Estrogen is known to increase vascular endothelial cell proliferation [14]. Therefore elevated estrogen exposure from hormone replacement therapy or oral contraceptives has the potential to promote the expansion of abnormal proliferative vascular lesions and subsequent thickening of the vasculature. At the molecular level how estrogen supports or promotes these atherosclerotic lesions is not clear. Therefore, the aim of this study was to investigate whether 17β-estradiol (E2)-induced ROS signaling is involved in the stimulation of the growth of endothelial cells.

## Methods

### Cell culture conditions and treatments

Human umbilical vein endotheilial cells (HUVECs) were obtained from American Type Culture Collection (Manassas, VA). Cells were grown in endothelial cell basal medium-2 (EBM-2) supplemented with EGM 2-MV Single-Quots (Cambrex/BioWhittaker, Walkersville, MD). Target tissue levels of 17β-estradiol (E2) are reported to range from 69.8 fmol/g to 679.9 fmol/g of tissue among postmenopausal women [15]. We will focus our study using concentrations of E2 (367 fmol and 3.67 pmol per ml medium) which are equivalent to physiological and pharmacological levels found in the target tissues. The effects of E2 treatments (367 fmol and 3.67 pmol/ml medium) on oxidant formation and DNA synthesis were studied under the following culture conditions: 70–80% confluent cultures were washed and serum starved in phenol red-free medium (mammary epithelium basal medium; Cambrex/BioWhittaker) for 3 h. Thereafter, the cells were treated with E2 for the indicated time periods. The effect of chemical inhibitors (rotenone, allopurinol, apocynin, DPI, L-NAME) and antioxidants (N-acetylcysteine and ebselen) was studied by pretreating the cells for 3 h prior to E2 exposure.

### Measurement of reactive oxygen species (ROS)

Cells were seeded at a concentration of 20 × 10^3 ^cells per well in black 96-well plates. Cells were pretreated with various antioxidants or inhibitors in Hank's balanced salt solution (HBSS) followed by incubation with 10 μM of 2'7'-dichlorofluorescin-diacetate (DCFH-DA) (Molecular Probes, Oregon) for 15 min. DCFH-DA stock solution was diluted at a 1:1 ratio with Pluronic^® ^F-127 (20% w/v). Cells were then rinsed with HBSS followed with various treatments described in the figure legends. DCFH-DA is a non-fluorescent cell-permeable compound, which is acted upon by endogenous esterases that remove the acetate groups generating DCFH. In the presence of intracellular ROS, DCFH is rapidly oxidized to the highly fluorescent 2', 7'-dichlorofluorescein (DCF). The oxidative products were measured with a Tecan Genios microplate reader using 485 nm and 535 nm as excitation and emission filters, respectively. In addition, DAF-FM diacetate (4-amino-5 methylamino-2',7'-difluorofluorescein diacetate) and dihydroethidium (Molecular Probes) were used to specifically measure nitric oxide and superoxide anion.

### BrdU cell proliferation assay

HUVECs were plated in 96-well plates at a density of 7,500 cells/well and incubated at 37°C with 5% CO_2 _overnight for attachment. In each experimental set, cells were plated in triplicates and were washed and incubated for 3 h prior to treatments in serum-free mammary epithelium basal medium devoid of phenol red. Cells were treated with E2 in the presence or absence of antioxidants for 18 h. Cellular proliferations were measured by colorimetric immunoassay based on BrdU incorporation into the cellular DNA by following the instructions recommended by the vendor (Cell Proliferation ELISA, BrdU Kit; Roche Molecular Biochemical, Indianapolis, IN). Briefly, cells were pulsed with BrdU labeling reagent for 3 h followed by fixation in FixDenat solution for 30 min at room temperature. Thereafter, cells were incubated with 1:100 dilution of anti- BrdU-POD for 1 h at room temperature. Finally, the immunoreaction was detected by adding the substrate solution and the color developed was read at 370 nm with a Tecan Genios microplate reader.

### Statistical analysis

Results are expressed as mean ± S.D. Differences between means were evaluated by two-tailed Student's *t*-test. ANOVA was used to determine differences between groups.

## Results

### E2-induced ROS production

**Figure 1:**
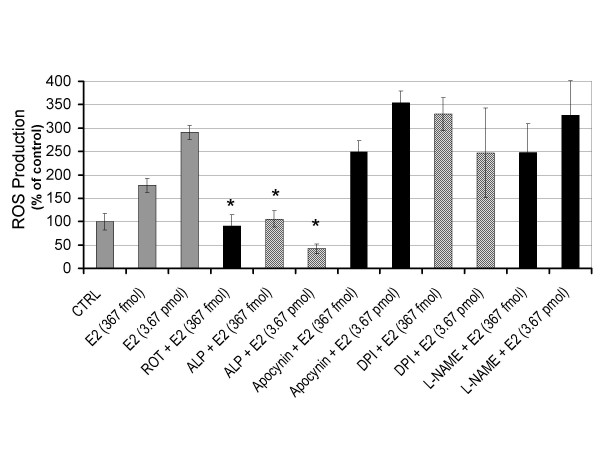
Estrogen stimulates the formation of reactive oxygen species in endothelial cells. Intracellular reactive oxygen species (ROS) production was determined by measuring the intensity of DCF. Human umbilical vein endothelial cells showed a dose dependent increase in oxidant production after 5 min of 17β-estradiol (E2) exposure. Pretreatment with the mitochondrial chemical inhibitor, rotenone (ROT), significantly reduced the level of E2 stimulated oxidant formation. Xanthine oxidase inhibitor, allopurinol (ALP), also showed a significant reduction of oxidant production in E2 treated endothelial cells. Co-treatments with the NADPH oxidase inhibitors, apocynin and DPI, did not decrease the production of oxidants in E2 treated cells. An inhibitor of nitic oxide synthase, L-NAME, also did not show any inhibitory effect on oxidant production in E2 treated endotheial cells. Concentrations of chemical inhibitors were as follows: rotenone (2 μM), allopurinol (50 μM), apocynin (30 μM), DPI (2.5 μM), and L-NAME (50 μM). Data from three independent experiments are presented as ROS production with controls set at 100% (± SD). Values that are significantly different from E2 treatment alone (*P *< 0.05) are indicated with an asterisk (*).

**Figure 2:**
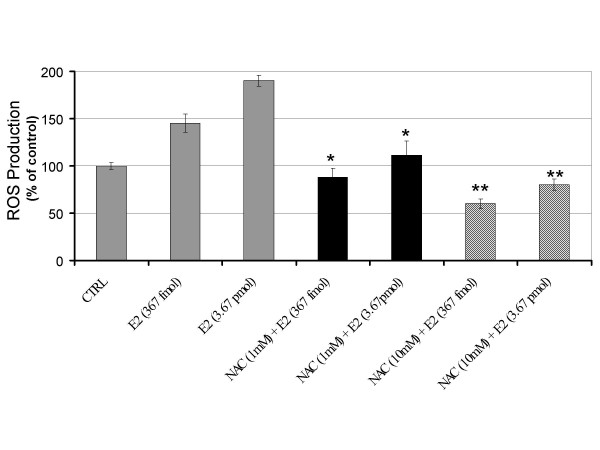
Antioxidant N-acetylcysteine suppresses the level of estrogen-induced ROS in endothelial cells. DCF intensity was measured in endothelial cells co-treated with antioxidants to verify E2-induced oxidant production. Human umbilical vein endothelial cells pretreated with the antioxidant N-acetylcysteine (NAC) showed a significant reduction of E2-induced oxidants to the level of control. Data from three independent experiments are presented as ROS production with controls set at 100% (± SD). Values that are significantly different from E2 treatment alone (*P *< 0.05) are indicated with an asterisk (*).

**Figure 3:**
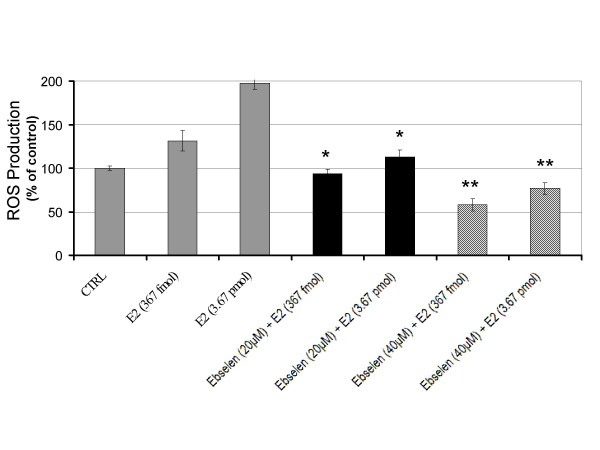
Glutathione peroxidase mimic inhibits the formation of estrogen-induced reactive oxygen species. Pretreatment with the glutathione peroxidase mimic ebselen showed a significant reduction of E2-induced oxidants in endothelial cells. Data from three independent experiments are presented as ROS production with controls set at 100% (± SD). Values that are significantly different from E2 treatment alone (*P *< 0.05) are indicated with an asterisk (*).

To evaluate whether E2 can trigger the rapid formation of intracellular oxidants, HUVECs were seeded in 96-well plates and pre-incubated with the redox-sensitive fluorescent dye DCFH-DA. Estrogen treated HUVECs showed a dose-dependent rapid production of ROS when exposed to E2 (Figure 1). The similar increase was not observed in cells exposed to vehicle. The E2-induced ROS formation continued beyond 90 min (data not shown). This is in agreement with our previous finding in breast epithelial cells [16]. To identify the source of intracellular ROS, we tried to suppress E2 triggered ROS production using selective chemical blockers. In HUVECs that were co-treated with the mitochondrial complex I inhibitor rotenone, we observed a significant reduction of E2-induced ROS to the level of control (Figure 1). The level of ROS in rotenone only treated cells was similar to cells exposed to only vehicle (data not shown). We also observed a significant decrease in E2-induced oxidants when HUVECs were co-treated with the xanthine oxidase inhibitor allopurinol (Figure 1). Allopurinol only treated cells showed a level of ROS equal to basal intracellular levels of vehicle alone (data not shown). Thus, both rotenone and allopurinol counteracted the increase of E2-induced ROS without affecting the basal intracellular level. Our results excluded the involvement of NAD(P)H oxidase since the selective inhibitors diphenylene iodonium (DPI) and apocynin did not prevent E2-induced oxidant formation. In addition, E2 stimulated ROS production remained unaffected in the presence of the nitric oxide synthase blocker L-NAME (Figure 1). To confirm the production of ROS by E2, we pretreated HUVECs with the antioxidant N-acetylcysteine (NAC) and the glutathione peroxidase mimic ebselen for 3 h prior to estrogen treatment. A significant reduction in E2-induced ROS was shown by the antioxidant NAC (Figure 2) and ebselen (Figure 3). Based on these findings we conclude that E2 can stimulate a rapid production of intracellular ROS in endothelial cells by mitochondria and xanthine oxidase.

### Antioxidants suppress E2-induced DNA synthesis

**Figure 4:**
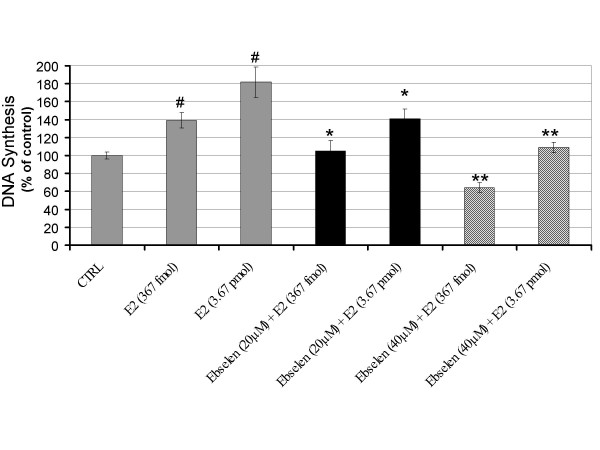
Glutathione peroxidase mimic ebselen inhibits estrogen-induced DNA synthesis. Cellular proliferation in the control and the treated cultures was measured by colorimetric immunoassay based on BrdU incorporation into the cellular DNA. Pretreatment with the glutathione peroxidase mimic ebselen showed a significant reduction of E2-induced DNA synthesis in endothelial cells. Data from three independent experiments are presented as ROS production with controls set at 100% (± SD). Values that are significantly different from E2 treatment alone (*P *< 0.05) are indicated with an asterisk (*). Significant increases in DNA synthesis by E2 (*P *< 0.05) are indicated by (#).

**Figure 5:**
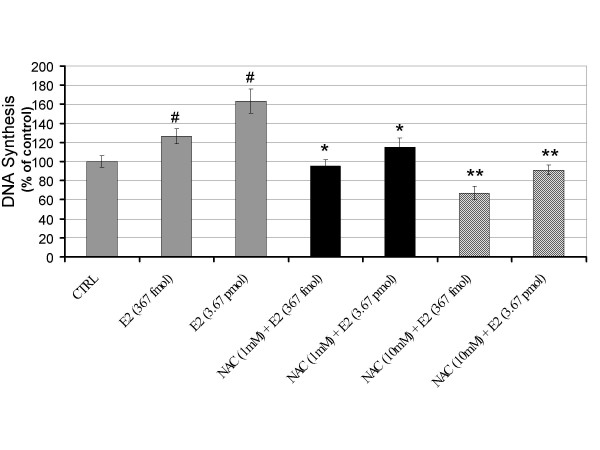
Antioxidant N-acetylcysteine blocks estrogen-induced DNA synthesis. Human umbilical vein endothelial cells pretreated with the antioxidant N-acetylcysteine (NAC) showed a significant reduction in E2-induced DNA synthesis. Data from three independent experiments are presented as ROS production with controls set at 100% (± SD). Values that are significantly different from E2 treatment alone (*P *< 0.05) are indicated with an asterisk (*). Significant increases in DNA synthesis by E2 (*P *< 0.05) are indicated by (#).

We have shown that E2-induced ROS act as signal-transducing messengers that control the early G_1_/S transition of G_0_-arrested estrogen dependent cells [17]. Therefore, we tested the influence of antioxidants on E2-induced DNA synthesis in vascular endothelial cells which is not considered to be an estrogen dependent tissue. E2-induced DNA synthesis at 18 h was evaluated by BrdU incorporation. E2 produced a significant 2-fold induction of BrdU incorporation (Figure 4). The similar increase was not observed in cells exposed to vehicle. We also evaluated the influence of the glutathione peroxidase mimic ebselen on E2-induced DNA synthesis. Cotreatment with ebselen (20 μM) significantly inhibited E2-induced DNA synthesis compared to E2 alone. This inhibitory effect was shown to be dose-dependent and suppressed E2-induced DNA synthesis by as much as 60% (Figure 4). The co-treatment of antioxidant NAC (1 mM) significantly decreased E2-induced DNA synthesis by as much as 100% (Figure 5). The NAC (1 mM) cotreatment with E2, which is equal to the basal control level, did not inhibit DNA synthesis which showed that NAC completely counteracted the E2-induced BrdU incorporation without affecting the basal levels of DNA synthesis. The inhibitory effect of NAC on E2-induced DNA synthesis was shown to be dose dependent.

## Discussion

Until recently, only nuclear ER signaling has been considered to be the major mechanism for regulating the growth of endothelial cells. High concentrations of E2 (10 μM) have been shown to act as antioxidants in vitro [18]. In contrast, our study used physiological concentrations of E2 (367 fmol and 3.67 pmol per ml medium) which do not act as antioxidants. Here we present data leading to the major novel findings that: (i) physiological concentrations of E2 trigger a rapid production of intracellular ROS in endothelial cells and (ii) E2-induced DNA synthesis is mediated by ROS signaling in endothelial cells. In our model, cells were blocked at the G_1_/S phase boundary by serum starvation and then pushed into S phase by the addition of estrogen. We demonstrated that the antioxidants ebselen and NAC block E2-induced DNA synthesis or S phase progression. Like several growth factors such as platelet-derived growth factor, epidermal growth factor, and nerve growth factor that are known to stimulate ROS and cell growth [19], our findings suggest that this underlying mechanism of cell growth is also shared with estrogen. Furthermore, the antioxidants NAC and ebselen, which are not ER antagonist, prevented E2-induced ROS mediated DNA synthesis and suggests that this signaling mechanism does not rely on ER genomic signaling. The conventional paradigm of estrogen action is based on binding to its receptors, ERα/β, which initiates transcription by binding to estrogen response elements of genes involved in cell growth. Discrepancies between the binding affinity of various estrogens to the ER and their growth potency both in vitro and in vivo have been reported [20,21]. Although selective ER modulators such as tamoxifen and antiestrogens such as ICI 182,780 prevent the growth of estrogen dependent cells, the contribution of other mechanisms cannot be ruled out as these compounds also block metabolism and redox cycling of estrogen, and are free radical scavengers [22].

Endothelial cells could conceivably generate ROS from an NAD(P)H oxidase system, from xanthine oxidase or from mitochondria. A variety of endothelial cell subtypes express NAD(P)H oxidase, and this system has been implicated in the signaling activated during mechanical strain [23]. Since we have shown the dependence of E2-induced mitochondrial ROS on the cytoskeleton [24], we proposed that E2 could produce mitochondrial ROS via the cytoskeleton because it has been shown to occur in mechanical strained endothelial cells [25]. Several studies have concluded that NAD(P)H oxidase is involved, on the basis of the observation that strain-induced changes were inhibited by diphenylene iodonium (DPI) [26,27]. However, the flavoprotein inhibitor DPI also blocks virtually all cellular oxidase systems, including mitochondrial complex I, nitric oxide (NO) synthase, and xanthine oxidase [28]. Therefore, the inhibition by DPI is not specific for NAD(P)H oxidases. Furthermore, NAD(P)H oxidase activity has been shown not to increase while hydrogen peroxide levels did increase in cyclic strained endothelial cells [29]. This suggest that NAD(P)H oxidase may not be responsible for E2-induced ROS in endothelial cells.

To identify the source of intracellular ROS, we tried to suppress E2-induced ROS production using selective chemical inhibitors (Fig. 1). The doses of pharmacological inhibitors used in this study have been demonstrated to be the lowest dose necessary to inhibit fluorescence in unstrained cells [30]; these doses coincided with a 10-fold increase from the dose reported to inhibit 50% of enzyme activity for the targeted enzyme systems (apocynin for NADPH oxidase, allopurinol for xanthine oxidase, L-NAME for endothelial NO synthase (eNOS), and rotenone for mitochondrial complex I). Our results excluded the involvement of NAD(P)H oxidase because apocynin, a more specific inhibitor compared to DPI [31], was ineffective in preventing ROS production. Since endothelial cells release NO in response to estrogen activation of eNOS which results in vasodilation [32] and NO can potentially contribute to the oxidation of DCFH (43); we evaluated the participation of reactive nitrogen species (RNS) in the response to estrogen by inhibiting NO synthesis with L-NAME. E2 stimulated DCFH oxidation remained unaffected in the presence of the NOS inhibitor L-NAME, instead we observed an increase in ROS production. Pretreatment with rotenone, a specific blocker of mitochondrial complex I, completely abolished E2-induced ROS. Endothelial cells co-treated with the xanthine oxidase inhibitor allopurinol also showed a dramatic decrease in E2-induced ROS. Together this data suggests that the both mitochondria and xanthine oxidase are the source of ROS in estrogen treated vascular endothelial cells. A possible mechanism for mitochondrial ROS formation by E2 is via the cytoskeleton. The ligation of α5β1 integrins at the plasma membrane and reorganization of the actin cytoskeleton has been shown to mediate ROS production through the activation of Rac-1 [33]. Whether estrogen can bind to integrins is not known. Alternatively, E2 binding to a membrane estrogen receptor could initiate the signal to mitochondria via the cytoskeleton. More specifically, activation of Rac-1 may modulate voltage dependent anion channel activity via the cytoskeleton leading to a rise in mitochondrial membrane potential and ROS formation [34,35].

To date, the specific, individual ROS that is most relevant to vascular signaling pathophysiologically is yet identified. Nevertheless, selectively overproducing or removing hydrogen peroxice (H_2_O_2_) significantly altered atherogenesis in animal models. Given that the DCFH probe is more sensitive toward oxidation by H_2_O_2 _than superoxide anion [36] and based on our results which show an increase in E2-induced ROS production (Figure 1) that can be blocked with H_2_O_2 _scavenging compounds NAC (Figure 2) and ebselen (Figure 3); the identity of the E_2_-induced oxidant appears to be H_2_O_2_. Our data is corroborated by studies showing that H_2_O_2 _increases, while antioxidants such as catalase, sodium pyruvate, and superoxide dismutase decrease HUVECs cell growth [37]. In experimental animals, selectively overproducing or removing H_2_O_2 _significantly altered atherogenesis [38]. We previously showed that E2 exposure increases the growth of macrophages and the secretion of the pro-inflammatory cytokine TNF-α [10,39]. Taken together, these data suggest that endothelial cells produce ROS in response to E2 or indirectly in response to E2-induced cytokines. Atherosclerotic lesions have been proposed to occur as a result of the monoclonal expansion of a mutated vascular cell [40]. Thus, E2-induced ROS in endothelial cells may be an underlying mechanism for the development of vascular lesions.

## Conclusion

In summary, we have shown that E2 exposure of HUVECs stimulates the rapid production of intracellular ROS that is involved in signaling endothelial cell growth. It appears that the early E2 signaling does not require ER mediated genomic signaling because we can inhibit E2-induced growth by antioxidants. The results from this study have major implications in understanding the role of estrogen in the development of vascular lesions which is highly relevant to the cardiovascular health of individuals susceptible to harm from elevated estrogen exposure. Findings of this study may further expand research defining the underlying mechanism of how estrogen may promote vascular lesions. It also provides important information for the design of new antioxidant-based drugs or new antioxidant gene therapy to protect the cardiovascular health of individuals sensitive to estrogen.

## Abbreviations

ROS, reactive oxygen species; E2, 17β-estradiol; ER, estrogen receptor; HUVECs, Human umbilical vein endotheilial cells; DCFH-DA, 2'7'-dichlorofluorescin-diacetate; H_2_O_2_, hydrogen peroxide; nitric oxide, NO; RNS, reactive nitrogen species; DPI, diphenylene iodonium; NAC, N-acetylcysteine;

## Competing interests

The author(s) declare that they have no competing interests.

## Authors' contributions

The author has contributed to the design of the study, the data analysis, and the writing of the manuscript.

## Pre-publication history

The pre-publication history for this paper can be accessed here:


